# Action by the Great Hospitals

**Published:** 1919-03-22

**Authors:** 


### THE STUDY OF PREVENTIVE MEDICINE.

### Action by the Great Hospitals.

The termination of hostilities and the return of our trained scientists after the invaluable experience gained in various theatres of war open up a field of labour in the development of preventive medicine which must be of inestimable value in the future, and it is gratifying to find that the authorities of our hospitals, both professional and lay, are prepared to shoulder the responsibility which lies before them.

We know that the staffs of great hospitals like St. Bartholomew's, St. Thomas's, Guy's, and, as we now learn, the London, have schemes under consideration which they are prepared to put into full working order directly they see their way clearly to obtain the necessary financial support. To a large extent, this support must come from State funds, and we trust that the record of work done by these institutions in the past has been adequate to secure the confidence of the Government departments which must regulate the disposal of these monies. It is generally believed that the plans of all are conceived on the same principle as those set out by Lord Knutsford in his speech to the Governors of the London Hospital.

The clinical instruction which has been given in the wards of hospitals by the distinguished members of the voluntary staffs will, it is hoped, be retained in full, but it should be supplemented. We find that at St. Thomas's Hospital arrangements had been made in the early part of 1914 to open at least one ward as a beginning for the thorough investigation of certain diseases. It was proposed that the investigator who was to be in control of the Clinical Laboratory should take a certain class of case and deal with this thoroughly as to cause, treatment, and prevention. The Bacteriological Laboratory of this hospital was already fully prepared to undertake the work, but the services of the Director of the Pathological Laboratories were sought by the Army. He was released from his engagement and for three and a half years has rendered invaluable service in the East, and, at the present time, is giving a series of lectures at the hospital on the diseases met with in the sub-tropical war areas—malaria fever, blackwater fever, bacillary and amoebic dysentery, enterica and trench fever. Side by side with this work, the Chemical Pathologist was investigating the chemical aspect of various diseases. His services also were required by the military, and he has succeeded in establishing certain tests for cases of nephritis. Both these gentlemen were salaried officers of the hospital, devoting almost their entire time to the work. The latter has returned from abroad and, with an experienced, highly qualified assistant, is continuing the investigations, beds in a hospital ward being allotted to the treatment and strict observation of nephritis cases which have originated either overseas or in this country. It will be found that this is one of the diseases which must for years engage the attention of authorities in coping adequately with the problems it presents, and the Governors of the hospital have already consented to do all in their power to further these scientific investigations, which must be of the utmost value to the country.

Directly the Insurance Bill was passed and some provision made for the treatment of tuberculosis, the importance of providing adequate treatment in a general hospital was recognised at St. Thomas's, a whole-time medical officer, specially qualified for the work, being appointed to take charge of the department, and he has carried out these duties with notable success. The treatment of tuberculosis must be taken into the homes of the people, and the methods of St. Thomas's, as carried out by the Lady Almoner's Department, should be examined by all hospital authorities who are not familiar with them, as a pattern for consideration in dealing with their own problem. By this far-seeing action the Governors have retained the treatment of this disease and enabled the students to study thoroughly the diagnosis, treatment, and prognosis of the terrible scourge. In 1916 the Physico-Therapeutic Department was reorganised and established, specialists in massage, in Swedish remedial exercises, and in electro-therapy being appointed on a salary to take charge of these sections. The magnificent work done in the treatment of our wounded soldiers and civilian population is well known to all interested in the importance of developing this branch of medical science. St. Thomas's accepted the invitation of the London County Council to establish a department for the treatment of venereal disease. A whole-time medical officer, paid by the Governors, was appointed, together with assistants necessary to carry on the work. Special wards were provided, and a very successful and extremely useful department is now in full working order. It is found that none of these schemes interferes other than for good with the general clinical work of the hospital, but afford opportunities for students to gain special knowledge and training.

At a recent meeting of the Medical Society, great emphasis was laid on the importance of students gaining knowledge of the special treatment now given in physico-therapeutics, but the curriculum laid down by examining boards affords them little opportunity during the two years allotted to clinical studies of spending anything like adequate time on special branches of either medicine or surgery. As soon as they qualified all these young surgeons have, for the past four and a half years, willingly given their services in one or other branch of our forces.

The subjects of medical study are already so diverse that, if his natural ambitions are to be developed and his efforts wisely directed, the student needs and should have at his disposal the very finest possible tuition and advice. These can best be supplied by the appointment of highly qualified, fully trained men of distinction as chiefs

**The Study of Preventive Medicine**—(*continued*).

in the branches of medicine, surgery, and gynæcology to work in conjunction with the directors of both laboratories and special departments. This principle meets with almost general acceptance, but the problems which remain to be solved are the selection of the best men for these posts and the supply of adequate funds for their remuneration as whole-time officers, or as officers having a large part of their time specially allotted to carry out the purposes of their appointment. The details as to the assistants they require and of the co-ordination of their work with that of the general physicians and surgeons of the hospital have been very fully discussed, and are on the eve of a satisfactory solution.

It is essential that the public should give willing support to this great work, for surely it is infinitely better that disease should be prevented and the large sums which have been so generously given in the past no longer expended on the treatment of illness which might have been avoided.

